# Radiosensitization by the novel DNA intercalating agent vosaroxin

**DOI:** 10.1186/1748-717X-7-26

**Published:** 2012-02-27

**Authors:** Ira K Gordon, Christian Graves, Whoon J Kil, Tamalee Kramp, Philip Tofilon, Kevin Camphausen

**Affiliations:** 1 Radiation Oncology Branch, National Cancer Institute, 10 Center Drive, MSC 1002, Bldg 10 Rm. B3B100, Bethesda, MD 20892, USA

**Keywords:** Vosaroxin, SNS-595, Naphthyridine, Quinolone

## Abstract

**Purpose:**

Vosaroxin is a first in class naphthyridine analog structurally related to quinolone antibacterials, that intercalates DNA and inhibits topoisomerase II. Vosaroxin is not a P-glycoprotein receptor substrate and its activity is independent of p53, thus evading common drug resistance mechanisms. To evaluate vosaroxin as a clinically applicable radiation sensitizer, we investigated its effects on tumor cell radiosensitivity in vitro and in vivo.

**Methods:**

Vosaroxin's effect on post-irradiation sensitivity of U251, DU145, and MiaPaca-2 cells was assessed by clonogenic assay. Subsequent mechanistic and in vivo studies were performed with U251 cells. Cell cycle distribution and G2 checkpoint integrity was analyzed by flow cytometry. DNA damage and repair was evaluated by a high throughput gamma-H2AX assay. Apoptosis was assessed by flow cytometry. Mitotic catastrophe was assessed by microscopic evidence of fragmented nuclei by immunofluorescence. In vivo radiosensitization was measured by subcutaneous tumor growth delay.

**Results:**

50-100 nmol/L treatment with vosaroxin resulted in radiosensitization of all 3 cell lines tested with a dose enhancement factor of 1.20 to 1.51 measured at a surviving fraction of 0.1. The maximal dose enhancement was seen in U251 cells treated with 75 nmol/L vosaroxin (DEF 1.51). Vosaroxin exposure did not change cell cycle distribution prior to irradiation nor alter G2 checkpoint integrity after irradiation. No difference was seen in the apoptotic fraction regardless of drug or radiation treatment. The number of cells in mitotic catastrophe was significantly greater in irradiated cells treated with vosaroxin than cells receiving radiation only at 72 hr (*p *= 0.009). Vosaroxin alone did not significantly increase mitotic catastrophe over control (*p *= 0.53). Cells treated with vosaroxin and radiation maintained significantly higher gamma-H2AX levels than cells treated with vehicle control (*p *= 0.014), vosaroxin (*p *= 0.042), or radiation alone (*p *= 0.039) after 24 hr. In vivo tumor growth delay was 1.5 days for vosaroxin alone (IV 10 mg/kg), 1.0 days for radiation (3 Gy) alone, and 8.6 days for the group treated with vosaroxin 4 hours prior to radiation.

**Conclusions:**

Vosaroxin enhanced tumor cell radiosensitivity in vitro and in vivo. The mechanism appears to be related to inhibition of DNA repair and increased mitotic catastrophe.

## Introduction

Topoisomerase II inhibitors are a diverse class of anti-cancer drugs that include the anthracyclines (doxorubicin and daunorubicin), etoposide and quinolones[1]. Anthracyclines have effective and broad-spectrum anti-tumor activity but their clinical utility is frequently limited by systemic toxicity (i.e. cardiotoxicity with doxorubicin) or drug resistance (frequently mediated by p-glycoprotein). Several topoisomerase II inhibitors are known to potentiate the effects of radiation on tumor cells although the mechanisms of radiation sensitization remain an area of research[2-4].

**Figure 1:**
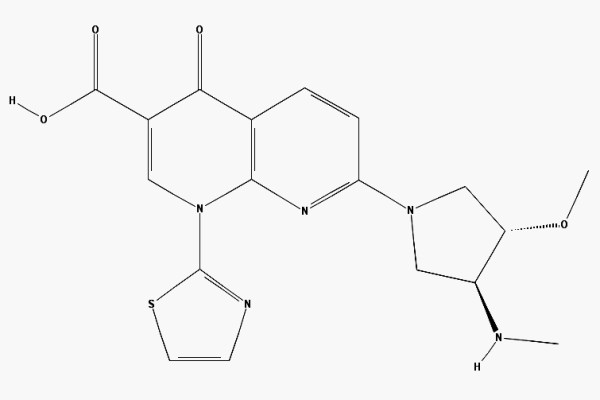
**Chemical structure of vosaroxin**.

Vosaroxin (Figure 1) is a naphthyridine analog structurally related to quinolone antibacterials, that intercalates DNA and inhibits topoisomerase II. Formerly known as voreloxin or SNS-595, it is the first drug in this class to be investigated as an anti-cancer agent. The drug's mechanism of action is similar to anthracyclines, involving intercalation of DNA and topoisomerase II inhibition, causing DNA damage in M and S-phase cells [5]. However, vosaroxin does not have known cardiotoxicity, is not a substrate for P-glycoprotein drug pumps, and has p53 independent activity. Vosaroxin has been shown to be active against various in vitro and in vivo tumor models including breast, bladder, pancreas, colon, ovarian, gastric, and lung cancer [6]. It has also shown synergistic activity with platinum agents, anthracyclines, antimetabolites, and targeted therapies in tumor models [6,7]. As an initial step in evaluating vosaroxin as a clinically applicable radiation sensitizer, we investigated the effects of vosaroxin on the radiosensitivity of a panel of human tumor cell lines. The data indicate that vosaroxin enhances tumor cell radioresponse in vitro and in vivo. Moreover, the mechanism appears to involve the inhibition of DNA double strand break repair.

## Materials and methods

### Cell lines and treatment

DU145 prostate carcinoma cells, MiaPaCa-2 pancreatic carcinoma cells, and U251 glioblastoma cells obtained from the ATCC were used for clonogenic assay experiments and U251 cells were used in subsequent experiments to investigate the mechanisms of radiosensitization. Cells were grown in DMEM (Invitrogen) supplemented with 10% fetal bovine serum and maintained at 37°C in a 5% CO_2 _atmosphere. Vosaroxin, provided by Sunesis, was reconstituted in DMSO (10 mmol/L) and stored in the dark at room temperature. For all studies, the working concentration of DMSO was < 0.1%. Cultures were irradiated at a dose rate of 2.28 Gy/min by a Pantak X-ray source.

### Clonogenic assay

Cultures were trypsinized to generate a single-cell suspension. A specified number of cells were seeded into each well of six-well tissue culture plates. Cells were allowed to attach for 6 hours followed by treatment with vehicle control or vosaroxin and were irradiated 16 hours later. Ten to fourteen days after seeding the cells, colonies were stained with crystal violet and the number of colonies containing at least 50 cells was determined. The surviving fractions were calculated and survival curves generated after normalizing for cytotoxicity from vosaroxin treatment alone.

### Cell cycle analysis

Evaluation of the cell cycle and G2-checkpoint integrity was performed by flow cytometry. Cells grown in 100 mm^2 ^culture dishes were exposed to vehicle control or vosaroxin for 16 hours prior to administration of 2 Gy or sham radiation. Cells were trypsinized 1, 3, 6, and 24 hours later, then fixed and stained per manufacturer's instructions with Cell Cycle Reagent and analyzed on an EasyCyte Plus flow cytometer (Guava Technologies, Hayward, CA). G2-checkpoint integrity was evaluated as previously reported by Xu, et al. [8,9] utilizing rabbit polycolonal antibody against phospho-H3 histone (Millipore) followed by staining with a goat anti-rabbit-FITC conjugated secondary antibody (Jackson ImmunoResearch) to distinguish mitotic cells.

### Apoptotic cell death

Apoptotic fraction was evaluated by flow cytometry using the Guava Nexin assay. Cells grown in 100 mm^2 ^culture dishes were exposed to vehicle control or vosaroxin, for 16 hours prior to administration of 2 Gy or sham radiation. Cells were trypsinized and stained 24 and 72 hours after radiation per manufacturer's instructions with Nexin Reagent to assess annexin V-PE as a marker of cells in early apoptosis and 7-AAD as an indicator of late apoptosis (Guava Technologies, Hayward, CA). Analysis was performed on an EasyCyte Plus flow cytometer. Positive controls were treated 24 hours with 0.5 μM staurosporine (Sigma).

### Mitotic catastrophe

The presence of fragmented nuclei was used to define cells undergoing mitotic catastrophe. Cells were grown on 4-well chamber slides. Cells were fixed with methanol for 15 minutes at -20°C, blocked with 1% BSA, and stained overnight at 4°C with mouse anti-α-tubulin antibody (Sigma) followed by staining with goat anti-mouse-Texas Red antibody (Jackson ImmunoResearch) 2 hours at room temperature. Nuclei were counterstained with DAPI (Sigma). Coverslips were mounted with VectaShield anti-fade solution (Vector Labs) and were visualized on a Leica DMRXA fluorescent microscope with a 20x objective (Wetzlar, Germany). Digital images were captured by a MicroPublisher 3.3 camera (QImaging, Surrey, Canada) and overlaid in Adobe Photoshop CS (San Jose, CA). Normal cells and cells in mitotic catastrophe were manually counted with the presence of nuclei fragmented with ≥ 2 lobes as the criteria for defining cells undergoing mitotic catastrophe. For each treatment condition 150-200 cells were scored.

### Electrochemiluminescent gamma-H2AX assay

A previously validated electrochemiluminescent assay was used to measure gamma-H2AX levels [10]. Cells were grown and treated on 150 mm^2 ^plates. Cells were exposed to vehicle control or vosaroxin, for 16 hours prior to administration of 2 Gy or sham radiation. 1, 6, and 24 hours later, cells were harvested and scraped into PBS, washed, and pelleted by centrifugation at 100 relative centrifugal force for 10 minutes. The PBS supernatant was discarded and the cell pellet was frozen at -80°C overnight. Cells were resuspended in lysis buffer of NaCl (500 mM), EDTA (2 mM), Triton X-100 (1%), sodium deoxycholate (1%), SDS (1%), Tris HCl (50 mM), NaF (10 mM), phosphatase and protease inhibitors (1 ×), and PMSF (2 mM). Proteins were solubilized by sonication and protein concentration was determined by Bradford assay. 5 μg of protein from each cell lysate was coated onto a 96-well plates and left overnight. Wells were blocked with 3% blocking solution, washed, and a sulfo-ester tag conjugated phospho-H2AX (Abcam) detection antibody was added in 1% blocking solution (1 μg/ml). Wells were washed thrice with TBS. A read buffer was added before analysis in an electrochemiluminescent imager (Meso Scale Discovery).

### In vivo subcutaneous tumor growth delay assay

Four to six week old, female, athymic NCr *nu/nu*, nude mice (NCI Animal Production Program, Frederick, MD) were used for all in vivo studies. Animals were caged in groups of 5 or less and were fed animal chow and water ad libitum. A single cell suspension (10 × 10^6^) of U251 cells was implanted on the lateral aspect of the rear leg. When tumors reached ≈ 100 mm^3 ^(*[L × W^2^]/2*) animals were randomized to four groups including untreated controls, vosaroxin (10 mg/kg IV into tail vein), irradiation alone (3 Gy), or vosaroxin + irradiation (10 mg/kg IV into tail vein + 3 Gy). Irradiation of tumors took place 4 hours after treatment with vosaroxin based on suspected drug metabolism in vivo, with animals restrained in lead jigs custom made by the Radiation Biology Branch of the National Cancer Institute. Tumors were measured three times per week until they reached ≥ 1000 mm^3^. Specific tumor growth delay was calculated for individual animals. The growth delay for the group was calculated as the mean number of days to reach 1,000 mm^3 ^in the treatment group minus the mean number of days for the control group to reach the same size. Group means ± standard error are reported. Each experimental group contained 6 animals. All animal studies were conducted in accordance with the principles and procedures outlined in the NIH Guide for the Care and Use of Animals.

### Statistical analysis

In vitro studies were subject to three independent experiments. Data is presented as mean ± SE. A Student's *t *test was used to compare sample means with a p value of < 0.05 considered significant.

## Results

To determine the effects of vosaroxin on the radiosensitivity of tumor cells, a clonogenic assay was performed. The previously reported average IC_50 _value for vosaroxin alone in 20 cancer cell lines was 322 nmol/L [6]. As drug exposure times are longer during clonogenic survival studies with radiation, concentrations from 50 nmol/L to 100 nmol/L of vosaroxin were used.

**Figure 2:**
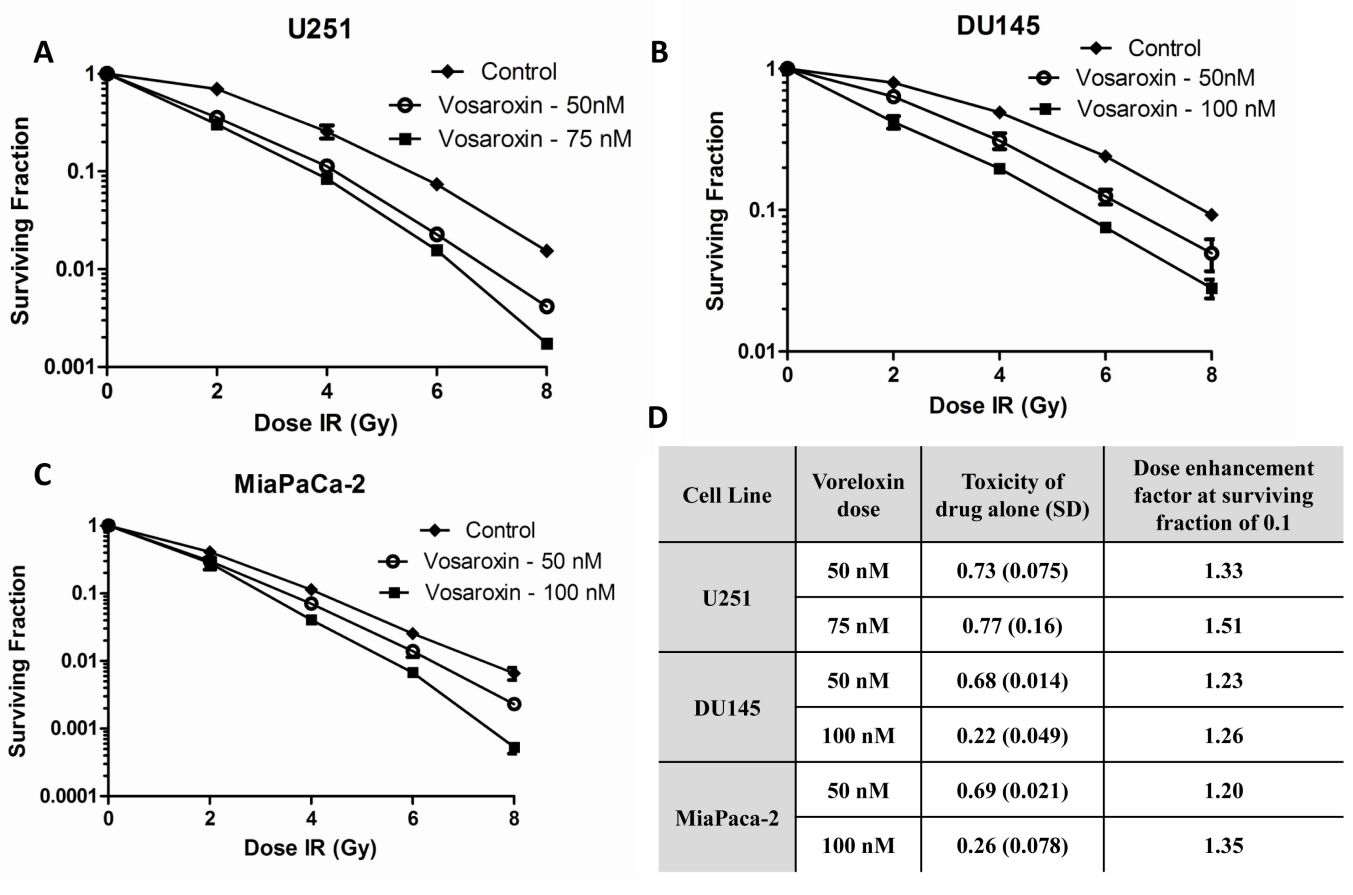
**Vosaroxin enhances radiation sensitivity in tumor cell lines**. The effects of vosaroxin on radiosensitivity by clonogenic assay. Cells were seeded as a single-cell suspension with a specified number of cells. After allowing cells time to attach (6 h), vosaroxin or the vehicle control was added at specified concentrations and the plates were irradiated 16 h later. Ten to fourteen days after seeding, survival curves were generated after normalizing for the cytotoxicity generated by vosaroxin alone. Figure shows U251 (**A**), DU145 (**B**), MiaPaca-2 (**C**), and a table showing cytotoxicity of vosaroxin alone and the radiation dose enhancement factor (DEF) for each cell line and drug dose combination (**D**). Data presented are the mean ± SE from at least three independent experiments.

Treatment of U251 cells with vosaroxin at 50 nmol/L and 75 nmol/L yielded a surviving fraction of 0.73 ± 0.075 and 0.77 ± 0.16 respectively which is an appropriate degree of cytotoxicity for evaluation in combination with radiation. In the combination protocol, 16 hours after drug was added, cells were irradiated and colony forming efficiency was determined 10-14 days later. This treatment resulted in a dose enhancement factor of 1.33 and 1.51 for 50 nmol/L and 75 nmol/L, respectively, at a surviving fraction of 0.10. The clonogenic assay was repeated with DU145 and MiaPaca-2 cells. These cell lines also showed radiosensitization with dose enhancement factors of 1.20-1.35 at 50-100 nmol/L. Cytotoxicity was found to be greater at 100 nmol/L than 50 nmol/L in these cell lines (surviving fraction of 0.22-0.26 vs. 0.68-0.69) in DU145 and MiaPaca-2. The survival curves and a table summarizing the results from the clonogenic assays are shown in Figure 2

**Figure 3:**
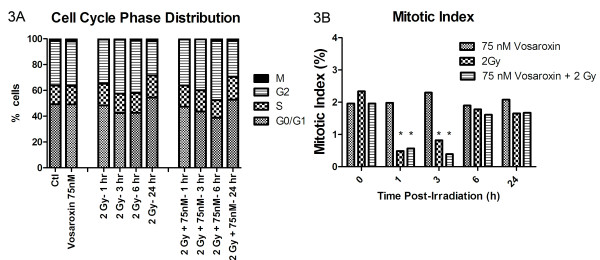
**Cell cycle distribution and mitotic index after irradiation is not altered by vosaroxin**. The percentage of cells in G0/G1, S, and G2/M phase of the cell cycle before and after radiation (2 Gy) with and without vosaroxin treatment (75 nmol/L). There was no difference in (3A) cell cycle distribution or (3B) mitotic index with vosaroxin treatment. *, *p *< 0.05 comparing mitotic index at 1 and 3 hours after radiation to baseline.

To begin to assess the cellular mechanisms or processes through which vosaroxin enhanced radiosensitivity, we focused on 75 nmol/L exposures in U251 cell lines due to the low cytotoxicity observed and the high dose enhancement factor. Cell cycle analysis was performed to determine if the drug exposure had resulted in accumulation of cells in a radiosensitive phase of the cell cycle. After a 16 hour incubation with 75 nM of vosaroxin alone, no difference in the ratio of cells in G1, S, or G2-M phase was seen (data not shown). Another potential source of radiosensitization is the abrogation of the G2 checkpoint after irradiation. To distinguish mitotic cells from G2 cells which both have 4 N DNA content and are not distinguished by propidium iodine staining, an antibody against phosphorylated histone H3 was used to detect mitotic cells as histone H3 is exclusively phosphorylated during mitosis, using the method of Xu [8,9]. As expected, the mitotic index (percentage of cells in mitosis), significantly decreased at 1 and 3 hours after radiation and then returned to baseline levels by 6 hours as a consequence of the normal G2 checkpoint activation initiated after radiation damage. In cells treated with the combination of vosaroxin and radiation, cell cycle distribution was the same as in the cells treated with radiation alone (significantly decreased mitotic index at 1 and 3 hours with return to baseline by 6 hours), indicating that there was no bypass of the G2 checkpoint (Figure 3). These results indicate that the radiation sensitization induced by vosaroxin is neither due to drug-induced alterations in cell cycle distribution at the time of irradiation nor due to abrogation of the G2 checkpoint after irradiation.

To provide insight into the mechanism of radiosensitization, we then determined the mode of cell death. At micromolar doses, vosaroxin has been shown to cause apoptosis in several cancer cell lines [5,11]. To determine if the 75 nM concentration of vosaroxin used in this study was inducing apoptosis after radiation, flow cytometry was performed. Less than 2% of cells in all treatment groups were apoptotic at 24 and 72 hours with no difference seen between untreated controls and cells treated with 75 nmol/L vosaroxin, 2 Gy, or the combination of 2 Gy and 75 nmol/L vosaroxin (data not shown).

**Figure 4:**
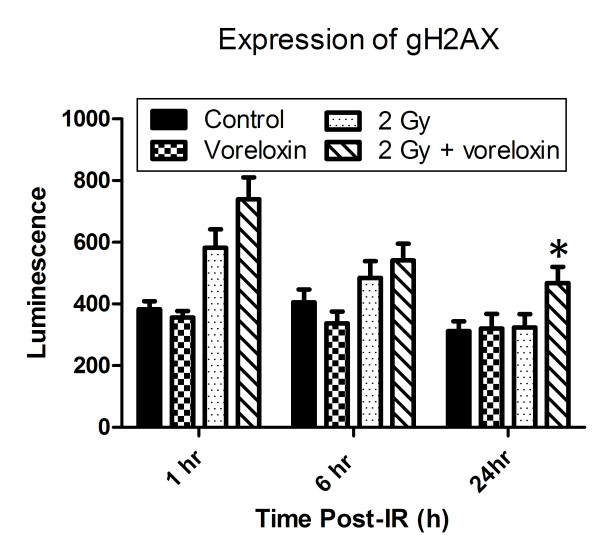
**Vosaroxin increases gamma**-**H2AX retention at 24 hours after irradiation**. A high throughput electrochemiluminescent assay to detect gamma-H2AX staining intensity was performed. Cells were plated in 100-mm cell culture dishes, allowed to attach (6 hr) and then incubated in vosaroxin (75 nmol/L) or vehicle containing media (16 hr) prior to irradiation (2 Gy). Cells were harvested at specified time points and stained with sulfo-ester tag conjugated phospho-H2AX detection antibody and luminescence was detected by an electrochemiluminescent imager. Data presented are the mean ± SE from at least three independent experiments. *, *p *< 0.05 comparing both radiation alone and vosaroxin alone treated cells to the combination treatment.

To assess the effects of vosaroxin treatment on DNA damage and repair, a high throughput electrochemiluminescent assay to detect gamma-H2AX levels in cells was used to measure DNA double strand breaks at 1, 6 and 24 hours (Figure 4). Vosaroxin alone caused no change in gamma-H2AX levels. At one hour, cells treated with radiation or the combination of vosaroxin and radiation had significantly higher gamma-H2AX levels compared to control or drug treated cells (< 0.001). At one hour, cells treated with both drug and radiation had higher mean gamma-H2AX levels than cells that were irradiated only (739.6 ± 70.7 and 582.4 ± 59.6) but this was not a significant difference (*p *= 0.11). At 6 hours, there was also no significant difference between cells treated with radiation alone versus vosaroxin and radiation (*p *= 0.47). As expected, at 24 hours, the cells treated with radiation alone had returned to baseline gamma-H2AX levels. However, the cells treated with vosaroxin and radiation maintained significantly higher gamma-H2AX levels than the cells treated with vehicle control (*p *= 0.014), vosaroxin (*p *= 0.042), or radiation alone (*p *= 0.039). The significant elevation of gamma-H2AX levels at 24 hours in the combination group compared to the other 3 groups suggests that vosaroxin inhibited DNA double strand break repair.

**Figure 5:**
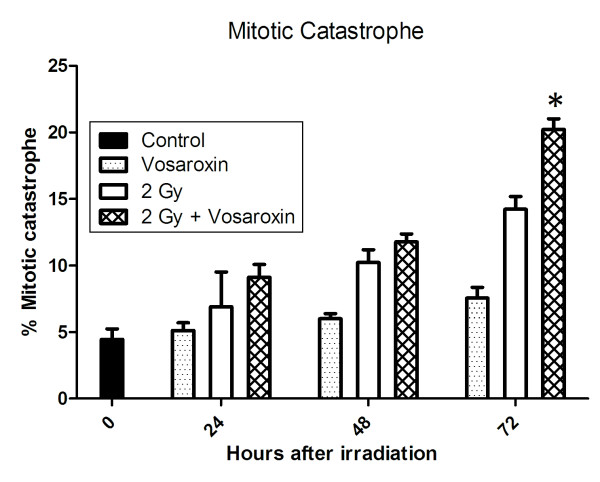
**Mitotic catastrophe after irradiation increases at 72 hours with vosaroxin treatment**. U251cells growing in chamber slides were exposed to vosaroxin (75 nmol/L) for 16 h, irradiated (2 Gy), and fixed at 24 h to 72 hours after irradiation for immunocytochemical analysis of mitotic catastrophe. Nuclear fragmentation (defined as the presence of two or more distinct lobes within a single cell) was evaluated in at least 150 cells per treatment per experiment. * = *p *< 0.05 comparing cells in the combination group compared to either drug or radiation alone groups at the same time point.

Since vosaroxin inhibits DSB repair and does not cause a significant increase in radiation induced apoptosis, we hypothesized that vosaroxin may induce radiosensitization through an enhancement of cells undergoing mitotic catastrophe. To assess mitotic catastrophe, the presence of fragmented nuclei was evaluated by immunofluorescence. As shown in Figure 5, the number of cells in mitotic catastrophe was significantly greater in irradiated cells treated with vosaroxin than cells receiving radiation only at 72 (*p *= 0.009) post-irradiation. There was no significant difference at 24 and 48 hours. Vosaroxin alone caused no significant increase in mitotic catastrophe over control cells at any time points (*p *= 0.53).

**Figure 6:**
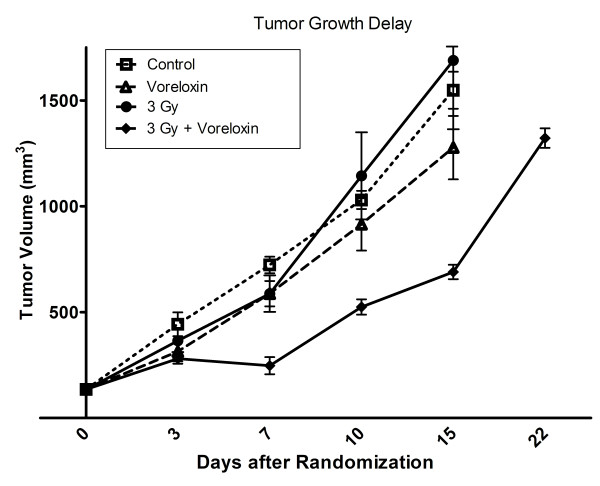
**Vosaroxin causes a greater than additive increase in tumor growth delay**. Mice were implanted with 1 × 10^6 ^U251cells on the lateral aspect of the rear leg. Tumors were randomized at 172 mm^3 ^into four groups and treated with either vehicle control, vosaroxin alone (10 mg/kg by tail vein injection), radiotherapy (3 Gy), vosaroxin/3 Gy. Tumors were measured three times per week and followed until they reached at least 1,000 mm^3^. Volumes were calculated using the formula (L × W × W)/2.

The in vitro data presented above indicate that vosaroxin enhances the radiosensitivity of U251 glioma cells and that the mechanism involves the inhibition of DSB repair. To assess the effect on tumors in an in vivo model, mice bearing U251 subcutaneous xenografts were randomized into four groups: vehicle, vosaroxin (10 mg/kg), irradiation alone (3 Gy), and vosaroxin and irradiation (10 mg/kg and 3 Gy). The average tumor sizes for the U251 tumors exposed to each treatment are shown in Figure 6. The average initial tumor volume was 172 ± 5 mm^3 ^with no significant differences in average initial size between groups. For each group, time to grow to 1,000 mm^3 ^(a 5-fold increase in tumor size) was calculated using the tumor volumes from each individual mice in each group (mean ± SE). The time for the tumor to grow from 172 to 1,000 mm^3 ^increased from 15.8 ± 0.8 days for vehicle-treated mice to 18.7 ± 4.3 days for vosaroxin-treated mice. Irradiated mice reached 1,000 mm^3 ^in 16.8 ± 3.5 days; mice treated with radiation and vosaroxin reached 1,000 mm^3 ^in 24.4 ± 1.0 days. The absolute growth delays (the time in days for tumors in treated mice to grow from 172 to 1,000 mm^3 ^minus the time in days for tumors to reach the same size in vehicle-treated mice) were 2.9 days for vosaroxin alone, 1.0 days for radiation alone, and 8.7 days for vosaroxin plus radiation therapy. Therefore the combination treatment group produced a tumor growth delay greater than the sum of the drug and radiation treatments alone (*p *= 0.004). These data indicate that vosaroxin enhances the radiation induced tumor growth delay of U251 xenografts.

## Discussion

This study demonstrated the enhanced radiosensitivity of human tumor cells after exposure to vosaroxin, an agent that acts as a DNA intercalator and an inhibitor of topoisomerase II. The potential of topoisomerase II as a target for radiosensitization has been previously suggested in studies with other agents in experimental tumor models.[2-4] Etoposide is a non-intercalating topoisomerase II inhibitor that enhances radiosensitivity due to effects on radiation repair and cell cycle [2,12]. The role of cell cycle in radiosensitization by topoisomerase inhibitors is complex. The mechanisms of radiation sensitization are important considerations because they influence the maximal cytotoxicity achieved in a time and sequence specific manner [4]. Radiation causes cells to arrest in G2/M phase which is when topoisomerase II typically functions as an important repair enzyme. Conversely, topoisomerase II inhibitors can cause G2 arrest which places cells in a relatively radiosensitive phase of the cell cycle [13]. However, in this study, G2 arrest was not seen at the doses of vosaroxin used. Therefore, this does not appear to be the mechanism of radiosensitization in this study.

The data presented here indicate that there was no significant initial increase in DNA damage based on gamma-H2AX levels at early time points after radiation suggesting no increase in the number of DNA-DSBs. However, at 24 hours after radiation, there was increased gamma-H2AX foci retention suggesting vosaroxin inhibits the repair of radiation induced DNA damage in U251 cells. Mechanistic conclusions made here are based only on one cell line and the mechanism of action of vosaroxin radiosensitization may differ among cell types. In addition, the potential for normal tissue radiosensitization will need to be considered in future work. A previous study evaluating the topoisomerase II inhibitors amrubicin and amrubicinol in lung adenocarcinoma showed similar enhancement of radiosensitivity to the results reported here with enhancement ratios of 1.38 and 1.57[3]. Similar to our findings, this study showed no increase in apoptosis when cells were irradiated and treated with topoisomerase inhibitors. The study found an increased proportion of necrotic cells after radiation and drug treatment with sub-additive increases in percent necrosis in the combination treated cells [3].

This data provides support for further evaluation of vosaroxin as a radiation sensitizer. As the first study to evaluate the radiation sensitizing properties of vosaroxin, this provides a basis for additional preclinical exploration of the radiosensitizing properties of vosaroxin. More thorough investigation and understanding of the specific molecular mechanisms leading to radiosensitization is warranted as these pathways were not identified in this study.

## Competing interests

The authors declare that they have no competing interests.

## Authors' contributions

IG performed the in vitro experiments, analyzed data and drafted the manuscript. CG and WK provided general assistance with the in vitro experiments. TM performed the animal experiments. PT and KC conceived of the study, analyzed data, assisted in preparation of the manuscript. All authors read and approved the final manuscript.
